# Emergency Medicine History and Expansion into the Future: A Narrative Review

**DOI:** 10.5811/westjem.2022.2.55108

**Published:** 2022-04-04

**Authors:** Martin R. Huecker, Jacob Shreffler, Melissa Platt, Dan O’Brien, Ryan Stanton, Terrence Mulligan, Jeremy Thomas

**Affiliations:** * University of Louisville School of Medicine, Department of Emergency Medicine, Louisville, Kentucky; † Central Emergency Physicians; ‡ University of Maryland School of Medicine, Department of Emergency Medicine, Baltimore, Maryland

## INTRODUCTION

Emergency medicine (EM) has advanced profoundly since its specialty recognition in 1979. As diagnosis and treatment changes, payment restructures and best practices evolve.^1^ We drive these changes, impacting hospital throughput and revenue to ensure quality emergency care. Our impact on the practice of medicine depends on a body of knowledge, the “biology” of emergency medicine.^2^ From 2000 to 2010 the number of emergency physicians (EP) increased more than in any other specialty.^3^ With estimates of over 48,000 EPs practicing in the United States currently and continued opening of new residency programs, multiple sources expect a 20–30% surplus of board-certified emergency physicians by 2030.^4^,^5^ Presciently, a 1997 paper by Holliman et al predicted that the supply of emergency doctors would equal demand in about 2020.^6^

In 2020, multiple EM organizations created a taskforce to study the projected EM workforce oversupply.^4^ The considerations address issues related to who practices emergency medicine (advanced practice providers, non-board-certified physicians) and who manages emergency clinicians (contract management groups, academic and hospital systems). Only two offer non-zero sum approaches: *broaden the umbrella to expand emergency physician scope of practice* and *expand the reach of emergency medicine to ensure that no community is left behind.4* This review aims to provide context for the workforce dilemma by describing the evolution of the scope of EM and possible future directions.

## EMERGENCY MEDICINE PAST AND PRESENT

Care for the acutely ill and injured patient traces its roots back thousands of years, but organized emergency medical care blossomed in the late 20th century (Figure). Emergency medicine became a medical specialty in response to several factors, chief among them the presence of patients with increased mobility who required unscheduled care that the current system could not accommodate (and increased financial support for these visits). A group of pioneers founded the Emergency College of American Physicians in 1968 in response to the need for physicians skilled in managing emergency patients.^7^ In 1970 Cincinnati opened the first EM residency. In 1976, the American Board of Emergency Medicine (ABEM) and the Society for Academic Emergency Medicine originated. After the American Board of Medical Specialties (ABMS) first voted 100 to 5 *against* our application for specialty status (1977), emergency medicine was approved as the 23^rd^ specialty in 1979.^8^,^9^

Leaders in EM have displayed innovation in approaching challenges related to patient care and organizational structure. *The Rape of Emergency Medicine* (1992) brought light onto the problem of patient and physician harm by management abuses.^10^ Decades later we contend with business interests often superseding patient care and education, with the explosion of hospitals and residency programs run by for-profit entities. We have struggled to maintain the commitment to quality training and patient care in a world of financial and economic motivation.

The field of EM adapted to medical and technological advances, resulting in diverse areas of focus that developed from the bottom-up into ABEM subspecialities: 1990s, Pediatric Emergency Medicine, Sports Medicine and Medical Toxicology; 2000s, Undersea and Hyperbaric Medicine, and Hospice and Palliative Care Medicine; 2010s, Anesthesiology Critical Care Medicine, Emergency Medical Services (EMS), Internal Medicine-Critical Care Medicine, Pain Medicine, and the focused practice designation in Advanced EM Ultrasonography.^11^ Subspecialities available to ABEM-certified physicians via other ABMS boards include Addiction Medicine, Brain Injury Medicine, Clinical Informatics, and Surgical Critical Care.

As the scope of EM continues to expand, our leaders can directly modulate the trajectory of the specialty. In 2011, Brian Zink wrote that EM “does not manage a specific disease, but the time dependent exploration of and intervention in the acute physical and/or psychological crises of humans.”^9^ Emergency physicians have increasingly become *the* expert acute diagnosticians. As the Model of the Clinical Practice of EM has expanded, from 22^12^ pages to 42,^13^ we retain our focus on the full spectrum of patient acuity, treating (a) critical, (b) emergent, and (c) lower acuity patients.^13^

Many factors have driven the successful growth of EM: the rise of hospital medicine; medical advances; improved transport; specialization of workforce; effective emergency treatments; efficiency and safety; and evidence-based medicine.^14^ Emergency medicine is now practiced in greater than 50 countries using many different models.^15^ Where our specialty has strong representation, outcomes improve for many illnesses: cardiac arrest; stroke; early analgesia; geriatric care; substance use treatment; psychiatric emergency care; and overall system efficiency.^14^,^16^ As Peter Cameron asserts, “EM is a specialty for the 21^st^ century^14^” because of our strengths in systems thinking and evidence-based medicine.

## FUTURE OF EMERGENCY MEDICINE

We agree with the ACEP Taskforce regarding the potential to “broaden the umbrella” and “expand the reach” – *not* to solve a workforce issue, but to embrace and deliberately shape the natural history of our specialty. Emergency physicians should continue to leverage our unique training to take ownership of undifferentiated patients. This includes “owning more of what we already do” (observation medicine, critical care, sports medicine, emergency psychiatric care, ultrasound, pediatrics, EMS, public health, etc) while also entering into new realms (proceduralist medicine, correctional care, pandemic preparedness, disaster medicine, rural medicine, pain/addiction, informatics and more).^4^ Below we offer a brief selection of possibilities for expansion and new frontiers in EM (Table 1).

### Education and Research

One cannot understate the importance of the academic development of EM with residency education, clinical quality assurance, and research. To discover and engage new frontiers, we need qualified individuals, valued as educators and researchers rather than solely for clinical revenue generation. Although EM researchers gain a small percentage of overall National Institutes of Health awards, our principal investigators received almost $90 billion from 2008 to 2017.^17^ Beyond growing and ensuring quality education in EM, academic emergency physicians should use their skills to educate and lead research in undergraduate and graduate medical education. We can participate in preclinical course instruction, simulation, academic administration, and clerkships to provide foundational EM knowledge to future physicians. Academic leaders in international EM devote time and energy to the development of EM training programs and health systems around the world.

### Public Health

Emergency physicians represent the first and sometimes only point of contact for large numbers of vulnerable individuals in the US healthcare system. The ED’s original role as the safety net in a complex medical system has sadly become more prominent and important. Simple public-health interventions in the ED include the standard screening for depression,^18^ domestic violence,^19^ and sex and human trafficking.^20^ The ED has for years attempted to reach individuals in need of immunization,^21^ and now can play a key role in COVID-19 vaccination.^22^

A 2009 EM publication called for more research, removal of barriers, innovation based on local needs, and legislation to improve incentives for large-scale community changes.^23^ Since then, research has covered screening for frailty and fall risk in the elderly,^24^ hypertension (with counseling/education),^25^ and motivation to provide the many material needs to address social determinants of health and disease (housing, food insecurity, unemployment, etc.).^26^

In the US, preventive healthcare in general receives embarrassingly scant incentives ($1 of preventive care for every $4 of treatment care).^27^ Focused training in public health represents an EM niche with almost unlimited value and reach. The COVID-19 pandemic has shown the potential for emergency physicians to successfully influence the public with health messaging and advising of government officials.

### Telehealth

The COVID-19 pandemic has brought telehealth to the forefront of medical innovation. Emergency medicine will continue to embrace new technology both in clinical practice and in training future physicians.^28^ Telehealth improves access to medical services and has rapidly increased in EDs throughout the US.^29^ While cost barriers exist, investment in these technologies will have clear downstream benefits to patients.^30^ Specific telehealth certification for EPs would help confront the legislative and litigative challenges. A 2015 systematic review of telehealth applications in the ED found “overwhelmingly positive” results in outcomes of technical quality, user satisfaction, clinical processes, throughput, and disposition.^31^

### Administration

Emergency physicians thrive in stressful clinical environments, managing unpredictability and making important decisions with limited data. We work closely with other physicians, understand patient flow into and out of the hospital, implement strategies for efficient patient care, understand technology and informatics, and can rapidly determine what does and does not work at individual and organizational levels. Who better to serve as leaders in healthcare than those with knowledge and experiences from the ground floor?

### Emergency Geriatric Medicine

As the number of US residents aged 65 years and older continues to grow, geriatric ED visits will continue to increase.^32^,^33^ Development and optimization of guidelines, physician training programs, and standards aimed at improving care for geriatric patients must be prioritized.^33^ Special training in Geriatric Emergency Medicine would incorporate clinical skills related to injury prevention/fall assessment, indwelling devices, medication management, delirium and dementia, and palliative care.^34^

### Emergency Medical Services

Well-established in medical direction roles with EMS, EPs now experiment with paramedicine to reach patients (for vaccination, buprenorphine treatment, preventive medicine) who have difficulty obtaining transport to healthcare services that are often completely absent in their communities. The role of EMS in addressing geographical healthcare disparities and social determinants of disease will expand in the next 10 years.

### Emergency Hospitalist Medicine

Internal Medicine-trained hospitalists manage hospital observation units, which optimize resource utilization.^35^ Despite overlap in clinical duties, internal medicine hospitalists and EM physicians do not regularly collaborate, missing the potential for enhanced patient care and even revenue generation.^36^ Emergency physicians and hospitalists could collaborate to manage short-stay patients and even train EM residents who have interest in observation or inpatient medicine.^37^ By improving patient care quality and streamlining hospital flow, we could solve the crowding issue from within our specialty.

### Rural Medicine

The physician oversupply issue predominates in urban areas, where 92% of EPs are employed, leaving rural EDs still largely underserved.^38^ Rural hospitals are closing at an alarming rate, potentially leaving 60 million people without adequate care.^39^ As clinicians who manage rural EDs age and retire, more rural areas will become “emergency physician deserts.”^38^ One-fifth of the US population resides in rural areas, but EM residents receive very little dedicated training.^40^ As Hill et al noted in the *Journal of Emergency Medicine*, “It is commonplace for programs not based in a Level I trauma center to send residents to an off-site rotation for trauma education. Why aren’t we doing the same for rural education?”^40^ Emergency medicine-primary care partnership models could address rural populations’ health needs.^41^ Increased presence of EPs in rural communities could alleviate our workforce concerns and treat the impending shortage of primary care physicians.

### Substance Use Disorder and Homelessness

As the healthcare gateway and often only point of contact, EPs should have optimal training to identify, treat, and refer patients with substance use disorder.^42^ Facing all-time highs in overdose deaths,^43^ EPs well connected with addiction services can combat the opioid and polysubstance use crises. Current fellowships in toxicology, pain control, and addiction medicine provide training for proficiency. The over three million homeless US residents^44^ are under-recognized in the ED and have unique needs unmet within the current models of emergency care.^45^ Having frequent encounters with homeless individuals,^44^ EPs can manage medical concerns while attempting to connect them with community resources.

### Wellness/Lifestyle Medicine

Emergency physicians are satirically portrayed as the “healthy doctors,” with multiple, often physically demanding habits and hobbies. We may be the ideal specialty to practice Lifestyle Medicine (which now has an active American Board of Lifestyle Medicine^46^). Entrepreneurial EPs already provide health optimization care in many locations in the country. Patients lost in the US “sick-care system” crave physicians who take care of their own health. Emergency physicians are clear candidates to assume wellness leadership roles in organizations (eg, Chief Wellness Officer).

## OBSTACLES

We were voted down on our first application for board status *and* our first application for primary board status. As EM continues its expansion, leaders will meet more challenges. Medicine represents a “wicked domain” where, unlike predictable rule-based domains (golf, chess), there is no causal structure, and we encounter tradeoffs. Generalists or “integrators” can step outside of the model more easily, grafting insights from one domain to use in another.^47^

Emergency medicine was not created by individual physicians, scientists, or health professionals. It was pulled into existence by the public itself and by the pathologies that for decades had fallen through the cracks in our system. Our success in the past 50 years reveals that EM is truly a different paradigm in medical practice and scientific thinking. Every new paradigm emerges from the pressure of unexplained, untreated, undescribed phenomena that the old one cannot explain.

We have now become the safety net not just for patients, not just for specialists, not just for the holes in our medical system, not just for the holes in our entire socioeconomic model, but also for the holes in our entire medical philosophy. By thoughtfully expanding into new niches, we broaden, rather than narrow, our scope. We discover obstacles to quality patient care and provide the missing components. As we overcome challenges, we must maintain balance: expand our abilities but on the foundation of fundamentals. It would be a mistake to let economics and workforce oversupply drive the evolution of our specialty.

## CONCLUSION

A career in EM provides a rewarding balance of risk, decision-making, and compassionate care for patients.^48^ As integrators constructing the future of EM, we can recognize limitations and look ahead with hope to an intelligent expansion. An early critic of EM said, “Emergency medicine is not a specialty, it is a location.”^49^ Let’s prove him wrong with a willingness to define ourselves not by the location in which we practice, but by the special, generalist care we deliver. While we contemplate expansion beyond the department, we must call for increased resources within the walls of the ED, with optimal staffing, reimbursement, and empowerment. Years ago, another critic said, “Emergency medicine is pointing in a very wrong direction which is bound to fail.”^49^ Whatever direction we are heading, let’s work together to guarantee success.

## Figures and Tables

**Figure:**
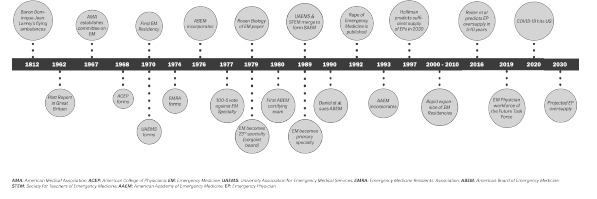
A timeline of Emergency Medicine Milestones.

**Table 1:** Emergency Medicine Subspecialties and New Frontiers.

Current accredited subspecialties	Current unaccredited subspecialties	New frontiers
*ABEM Subspecialties* Anesthesiology Critical Care Medicine Emergency Medical Services Hospice and Palliative Care Medicine Internal Medicine-Critical Care Medicine Medical Toxicology Pain medicine Pediatric Emergency Medicine Sports Medicine Undersea and Hyperbaric Medicine *Subspecialties other ABMS Boards* Addiction Medicine Brain Injury Medicine Clinical Informatics Surgical Critical Care *EM Focused Practice Designation* Emergency Medicine Ultrasonography	Administration/Operations/Quality and Safety Austere/Disaster medicine Emergency Imaging Geriatric Emergency Medicine Global/Population Health and Social Medicine Forensic Medicine Health Policy/Public Health Injury Control International Emergency Medicine Medical Education Neurovascular and Stroke Observation Medicine Occupational and Environmental Medicine Research Resuscitation Simulation Medicine Tactical Medicine Telemedicine Wilderness Medicine Women’s Health	Correctional Medicine Emergency Hospitalist Medicine Emergency Psychiatric Medicine Event Medicine Healthcare Innovation Patient Advocacy/Activism EM Proceduralist Rural Medicine Substance Use Medicine Wellness/Lifestyle Medicine

*ABEM*, American Board of Emergency Medicine; *ABMS*, American Board of Medical Specialties; *EM*, emergency medicine.
